# The relationship between previous pulmonary tuberculosis and risk of lung cancer in the future

**DOI:** 10.1186/s13027-022-00434-2

**Published:** 2022-05-07

**Authors:** Yongwei Qin, Yujie Chen, Jinliang Chen, Kuang Xu, Feifan Xu, Jiahai Shi

**Affiliations:** 1 Department of Pathogen Biology, Medical College, Nantong University, No. 19 Qixiu Road, Nantong, China; 2 Affiliated Nantong Hospital of Shanghai University, No. 500 Yonghe Road, Nantong, China; 3 Nantong Key Laboratory of Translational Medicine in Cardiothoracic Diseases, Nantong Clinical Medical Research Center of Cardiothoracic Disease, and Institution of Translational Medicine in Cardiothoracic Diseases, Affiliated Hospital of Nantong University, Nantong, China; 4 Department of Respiratory Medicine, The Second Affiliated Hospital of Nantong University, Nantong First People’s Hospital, No. 6 North Road Hai’er Xiang, Nantong, 226001 Jiangsu China

**Keywords:** Lung cancer, Tuberculosis, ROS, DNA damage, Cytokine, COX-2, CRP, Risk factor

## Abstract

Various investigations have expanded the views that tuberculosis is an important risk factor for lung cancer occurrence. Lung cancer originates from chronic inflammation and infection. It is becoming clearer that *Mycobacterium tuberculosis* (*M.tb*) in tuberculosis patients meticulously schemes multiple mechanisms to induce tumor formation and is indispensable to participate in the occurrence of lung cancer. In addition, some additional factors such as age, sex and smoking, accelerate the development of lung cancer after *Mycobacterium tuberculosis* infection. The clarification of these insights is fostering new diagnoses and therapeutic approaches to prevention of the patients developing from tuberculosis into lung cancer.

## Background

It is well known that lung cancer is now a public health issue as the leading cause of cancer-associated deaths in the world. The World Health Organization reported that about 1.76 million people worldwide die of lung cancer each year (https://www.who.int/news-room/fact-sheets/detail/cancer). It's the largest cause of death among men, and the third leading cause of cancer in women, after breast and colorectum cancer. Tuberculosis (TB) is another major public health problem worldwide, which is caused by *Mycobacterium tuberculosis*. About a third of people are infected with *Mycobacterium tuberculosis*, and about 1.4 million people died from TB in 2019 (https://www.who.int/news-room/fact-sheets/detail/tuberculosis). *Mycobacterium tuberculosis* infections can also trick the body's innate immune, which can be partially suppressed by *Mycobacterium tuberculosis* [1]. A weakened immune system is more likely to lead to disease, but whether it will elevate the risk of lung cancer is debatable.

There are many hypotheses that *M.tb* infection causes lung cancer, such as immune system suppression, DNA damage, and the production of inflammatory factors. The main hypothesis is that *Mycobacterium tuberculosis* causes chronic inflammation and thus promotes lung cancer. *Mycobacterium tuberculosis* is an intracellular organism, the ability of *Mycobacterium tuberculosis* to cause devastating lung disease in immune hosts allows it to spread effectively through the respiratory pathway in the population. Similarly, although the incidence of tuberculosis in cancer patients exposed to immune checkpoint inhibitors (ICI) is eightfold that of the general population, there is no striking difference in the risk of cancer patients developing tuberculosis based on different ICI exposures [2]. The causal relationship between tuberculosis and lung cancer is more widely accepted, however, recent studies of the cellular and molecular mechanism mediating this relationship remain unclear, and these are the main highlights of this review.

Epidemiological studies now support a causal relationship between the incidence of tuberculosis and lung cancer, providing epidemiological evidence for disease prevention and supporting national efforts to control tuberculosis. Tuberculosis was found to be significantly associated with adenocarcinoma, but not with squamous or small cell lung cancer. Although no causal mechanism has been demonstrated, the current study supports a direct relationship between lung cancer and tuberculosis, especially adenocarcinoma. Everatt R et al. have shown that tuberculosis patients have a higher risk of developing lung cancer [3–5]. A cohort study displayed that the incidence of lung cancer in tuberculosis patients (269 per 100,000 people per year) was significantly higher compared with healthy controls (153 per 100,000 people per year) [6]. Even though tuberculosis has been recognized as a likelihood factor for the development of lung cancer, the results of previous studies are still conflicting and uncertain due to the potential confusion between smoking and other comorbidities. Only Luo et al. found that tuberculosis was related to the development of lung cancer after exposure to tuberculosis for more than 20 years [7]. Moreover, Simsek suggested that we should consider *Mycobacterium tuberculosis* or NTM when treating lung cancer. Patients with tuberculosis may be misdiagnosed as lung cancer, and vice versa [8].

Based on a systematic review of the connection between TB and lung cancer risk, we found that there are many risk factors that the co-existence of pulmonary tuberculosis and lung cancer, such as smoking, age, gender and *Mycobacterium tuberculosis* infection. Smoking has long been a major cause of lung cancer and tuberculosis, so smoking is a leading suspect when researching the risk factors for tuberculosis developing into lung cancer [9]. There have been several studies investigating the relationship between lung cancer and tuberculosis, but very few studies have researched the ratio of age and gender in patients with tuberculosis and lung cancer [3, 10].

## Age, pulmonary tuberculosis and lung cancer

Several studies have investigated pulmonary tuberculosis as an age-related risk factor for lung cancer. The incidence of lung cancer and tuberculosis in the age of high incidence has a younger trend. According to the American Association of Thoracic Surgery, patients are considered as higher risk of lung cancer who are over 55 years old and have a smoking history of > 30 pack years. However, when looking at the coexistence of Tb and lung cancer, it was found that younger patients were more likely to develop the disease [11]. There is evidence in a literature using Meta-analysis indicated that Tuberculosis diagnosed at a young age is a risk factor for lung cancer, regardless of smoking history or other underlying disorders. This trend was more obvious among patients from high TB burden countries [12].

Wu et al. mentioned that TB which is crucial in the development of lung cancer in all age groups, particularly in younger patients [6]. And Soo Jeong An reported that younger patients with TB have a more substantial likelihood of lung cancer than older patients with pulmonary TB [13]. There is no evidence to describe the reason which needs amounts of clinical studies and data. So we recommend that younger patients with a history of TB should be evaluated with a series of tests for underlying lung cancer.

## Smoking as an independent effect factor

Smoking was an unconstrained risk element for both lung cancer and tuberculosis. So smoking is the first suspect in lung cancer induced by tuberculosis. The formation of the polycyclic aromatic hydrocarbon-DNA complex can lead to the induction of G-T transversion in TP53, which is due to DNA duplication of unrepaired DNA, which generates mutations at the site of DNA complex formation. At codon 157 of TP53, G-T transversion is common in smokers’ lung cancer, but not in never smokers [14]. Many researchers have conducted a series of studies to examine the effect of smoking (active smoking, passive smoking, and duration of smoking) on lung cancer caused by tuberculosis by controlling for age, alcohol, and other covariates. A question that needs to be asked, however, is whether such an experiment is rigorous. Hui-Ying Liang found that some of the drawbacks of the study were not fully controlled for environmental tobacco exposure, so it was inconclusive that smoking increased the risk of lung cancer with preexisting tuberculosis [15]. In a retrospective cohort study conducted in Xuanwei which included over 42,422 farmers and total of 2459 cases (5.8%) of LC were died, of which 246 cases (0.6%) were diagnosed with pulmonary TB. Lung cancer mortality was significantly higher in cases with pulmonary tuberculosis than in those without. There is no difference between men and women, those are always exposure to coal smoke from stoves used for cooking and heating [16].

The effect of smoking on the development of lung cancer from tuberculosis has become clear from various studies. Kunio Aoki showed that the risk of lung cancer in female TB patients with a low smoking rate was higher than that in male smokers [17]. This was influential in that it is reported that smoking did not make much difference between preexisting TB patients and non-TB patients who subsequently developed lung cancer. Interestingly, Hong et al. analyzed lung cancer risk in Korean lung cancer patients with and without a history of smoking, and reported that the risk was not associated with tuberculosis [18]. Park et al. proved that the history of tuberculosis is associated with an increased risk of lung cancer among COPD patients with a moderate burden of tuberculosis in Korean. COPD patients with a history of tuberculosis, especially those who have never smoked, may benefit from regular screening or evaluation for lung cancer development [19]. When information was complex, Seri Hong also stated there was no synergy between smoking and previous TB [18]. Smoking and tuberculosis were factors that affect the occurrence of lung cancer, but the influence of tuberculosis on the progression to lung cancer was negligible compared with that of smoking.

The relationship between lung cancer and tuberculosis caused by smoking is not certain. Singapore is the small, high-income country with the strictest ban on smoking, has the lowest smoking prevalence in the WHO Western Pacific Region. There has a moderate incidence of tuberculosis in Singapore. Since 1999, there has been a high proportion of non-small cell lung cancer. It is unknown whether or not lung cancer incidence in non-smokers has increased. It is also unclear what is driving cancer incidence in non-smokers [20].

## Gender and epidemiological studies

Various studies have been conducted on the male-to-female ratio of patients who previously had tuberculosis followed by lung cancer. According to World Health Statistics 2018, the smoking rate among Chinese aged 15 or older was 48.4% males and 1.9% females, which makes it difficult to control for confounding factors in gender studies. Then Kunio Aoki stated when he did his epidemiological study of the coexistence of tuberculosis and lung cancer, he found that females with tuberculosis have a higher risk of dying from lung cancer than males. There are several investigations that have attempted to reveal the phenomenon. Some of their findings are summarized in Table 1. It can be concluded that risks is inclined to higher in females than in males.

**Table 1:** Epidemiological reports on gender in tuberculosis and lung cancer

References	Country	Num. subjects	Finds
Steinitz [21]	Israel	15,400	LC RR was 7.98–9.76 for males aged 45–64 years and 10.8 for females aged 45 years and over
Horikoshi [22]	Japan	80,000	The MR was high at 4.88 for males and 9.69 for females
Takatorige [23]	England/Wales	3119	LC RR was 6.88 for males and 13.77 for females
Gao et al*.* [24]	China	30,373	The MR ratio was1.72 for males and 2.79 for females
Clemmesen [25]	Denmark	2790	LC RR was 2.58 for males without INH treatment and 4.55 for females

*LC* lung cancer, *RR* relative risk, *MR* mortality ratios

## Pulmonary tuberculosis and its mechanism

The course of tuberculosis is a chronic infection process, accompanied by lung tissue remodeling [26]. The typical pathological hallmark of this process is the generation of granulomas centered on mycobacteria, surrounded by myeloid cells and lymphocytes. Antigen-specific T lymphocytes produce interferon-γ and other cytokines. Activated macrophages produce more inflammatory cytokines, reactive oxygen species and nitrogen, prostaglandins, and proteases in an attempt to eliminate bacteria [27–29]. This process is like a double-edged sword, while eliminating tuberculosis bacilli, it also causes extensive damage to lung tissue. In the later period, the repetitive inflammatory injury and subsequent repair process of lung epithelial cells, which leads to abnormal growth factor activation and fibroblast aggregation, accompanied by the hyperplasia and metaplasia of lung epithelial cells. Recently, there have also been reports of the coexistence of tuberculous granuloma and lung cancer [30, 31]. These changes might represent the origin of the development of lung cancer [32].

## Cytokine of pulmonary tuberculosis and lung cancer

The development of cancer is a complicated process that can be facilitated or inhibited by changes in the microenvironment, and it has been shown that chronic inflammation can produce a microenvironment that is conducive to tumor development and progression [33]. According to the reports, *Mycobacterium tuberculosis* induces the secretion of inflammatory cytokines such as INF-γ, IL-1, IL-2, IL-12, and TNF, causing inflammation in lung tissues [26, 34]. The occurrence and development of tumors are closely related to cell hyperplasia and apoptosis, and inflammatory factors play an important role in this process. For example, TLR2 and IL-6, IL-17, and IL-22 were highly expressed in the serum of patients who suffer from tuberculosis and lung cancer [35]. Meanwhile, Ming Zhang found Silencing TLR2 promotes cell apoptosis [35]. Dheda K suggested that TNF and IL-6 may also up-regulate the expression of anti-apoptotic genes through the NF-κB pathway [34]. NF-κB signal pathway is actively responsible for a series of inflammatory respiratory diseases, including asthma, chronic obstructive pulmonary disease (COPD), pulmonary fibrosis, and lung cancer. It also exerts an important role in microbial infections, tuberculosis and COVID-19 [36–41]. These findings emphasize the role of NF-κB signaling in the pathogenesis of tuberculosis and lung cancer, and also play a bridge role for the pathogenesis of tuberculosis and lung cancer. Therefore, *Mycobacterium tuberculosis* may promote tumor development through inflammatory factors.

Many experiments have provided evidence that chronic *Mycobacterium tuberculosis* infection can lead to the development of lung squamous cell carcinoma in a mouse model and patients with preexisting tuberculosis [33]. Gupta shows that THP-1 infected with *Mycobacterium tuberculosis*, enhanced the invasion ability and induce morphology phenotype changes of human lung epithelium-derived lung adenoma A549 cell line from epithelial to mesenchymal during co-culture [42].

However, the exact molecular mechanism by which this happens is still unclear. The biological connection between tuberculosis and lung cancer predominantly focuses on the role of *Mycobacterium tuberculosis* infection and pulmonary fibrosis in the occurrence of lung cancer. Lung cancer can be promoted by repairing excessive and persistent *Mycobacterium tuberculosis* colonization and the resulting chronic inflammation and fibrosis [32, 43]. *Mycobacterium tuberculosis* infection can cause lung epithelial proliferation and damage [44, 45], and cytokines (pro-inflammatory cytokines: IL-1, IL-6, IL-17, IL-18, IL-22, TNF-α, IFN-γ; anti-inflammatory cytokines: IL-10, TGFβ, etc.) released by macrophages, DC, alveolar Type II pneumocyte can induce a cytokine storm and lung epithelial cell proliferation [46]. In addition to macrophages and lung epithelial cells, genetic and functional experiments have shown that neutrophils, activated T cells, mast cells, and eosinophils also cause malignant tumors by releasing extracellular proteases, chemokines and pro-angiogenic factors [47–51]. Matsuyama et al. proved that T lymphocytes of patients with active tuberculosis secreted high level of vascular endothelial growth factor (VEGF) than healthy controls. The secretion of this VEGF was inhibited by adding MHC class II antibodies. However, the addition of MHC class I antibodies has no inhibitory effect. VEGF is also secreted in the CD4^+^ T lymphocytes treated with purified protein derivative of tuberculin (PPD). The production of VEGF may be related to the antigen-specific immune response through CD4^+^ T lymphocytes in pulmonary tuberculosis. VEGF plays an important role in angiogenic and mitogenic properties. Therefore, VEGF may promote hypervascularity and promote the occurrence and development of lung cancer [52].

## Effector protein of *Mycobacterium tuberculosis* and lung cancer

**Fig. 1:**
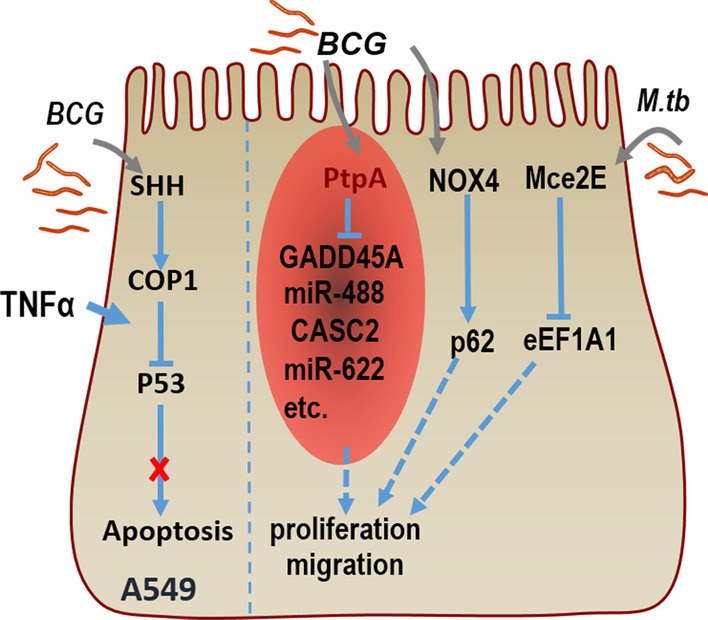
*M.tb* promotes proliferation and migration of A549 cells. BCG induces SHH signaling-dependent expression of COP1, which targets and degrades of P53, and it inhibits cell apoptosis of A549. PtpA-expressing BCG can promote proliferation and migration of A549 cells, partially through targeting GADD45A or ncRNA genes (such as miR-488, CASC2, and miR-622) which are related to tumor progression through regulating cell apoptosis, proliferation, and migration. NOX4-p62 might serve as a driving factor of the tumor microenvironment changed by tuberculosis fibrosis. Mce2E of *M.tb* can inhibit eEF1A1, which increase cell proliferation. These actions all promote proliferation and migration of A549

BCG activates SHH signal to express E3 ubiquitin ligase, (COP1)/RFWD2, which targets TNF-α reactive p53, thereby inhibiting cell apoptosis of A549 cell line. Classical nude mouse xenotransplantation studies have shown that BCG inhibits tumor clearance induced by TNF-α and promotes tumor formation. In conclusion, the results suggest that *Mycobacterium tuberculosis* inhibits apoptosis and promotes tumorigenesis [53]. Moreover, *Mycobacterium tuberculosis* effector protein PtpA can inhibit the transcription of GADD45A, which is a checkpoint protein-coding gene that encodes proteins involved in cell division, cell death and cell senescence in vitro and in the mouse xenograft model [45]. *Mycobacterium tuberculosis* Mce2E can inhibit K48-linked polyubiquitination of eEF1A1 to promote the protein stability of eEF1A1in the A549 cell line, leading to the enhancement of tumor cell proliferation [44]. Woo et al. showed that NADPH Oxidase 4 (NOX4) silencing impaired the malignant potential of A549 cells promoted by tuberculous pleural effusion. Mice with BCG pleura infection showed induced expression of NOX4 and increased tumorigenic potential of lung cancer compared with mice injected with PBS. The NOX4 knock-out (KO) mice showed reduced tuberculosis fibrosis and the potential to promote lung cancer metastasis, which indicates that the NOX4 signaling axis regulated by tuberculous fibrosis may lead to enhanced tumorigenic potential [54] (Fig. 1). In addition, a retrospective cohort study performed in Latin American displayed MTB DNA sequence IS6110 identified in the nuclear area of tumor cells in the lung [55]. These findings provide several evidences for a causal link between lung cancer and mycobacterium secreted protein and its DNA.

## Species of pulmonary tuberculosis and lung cancer

**Fig. 2:**
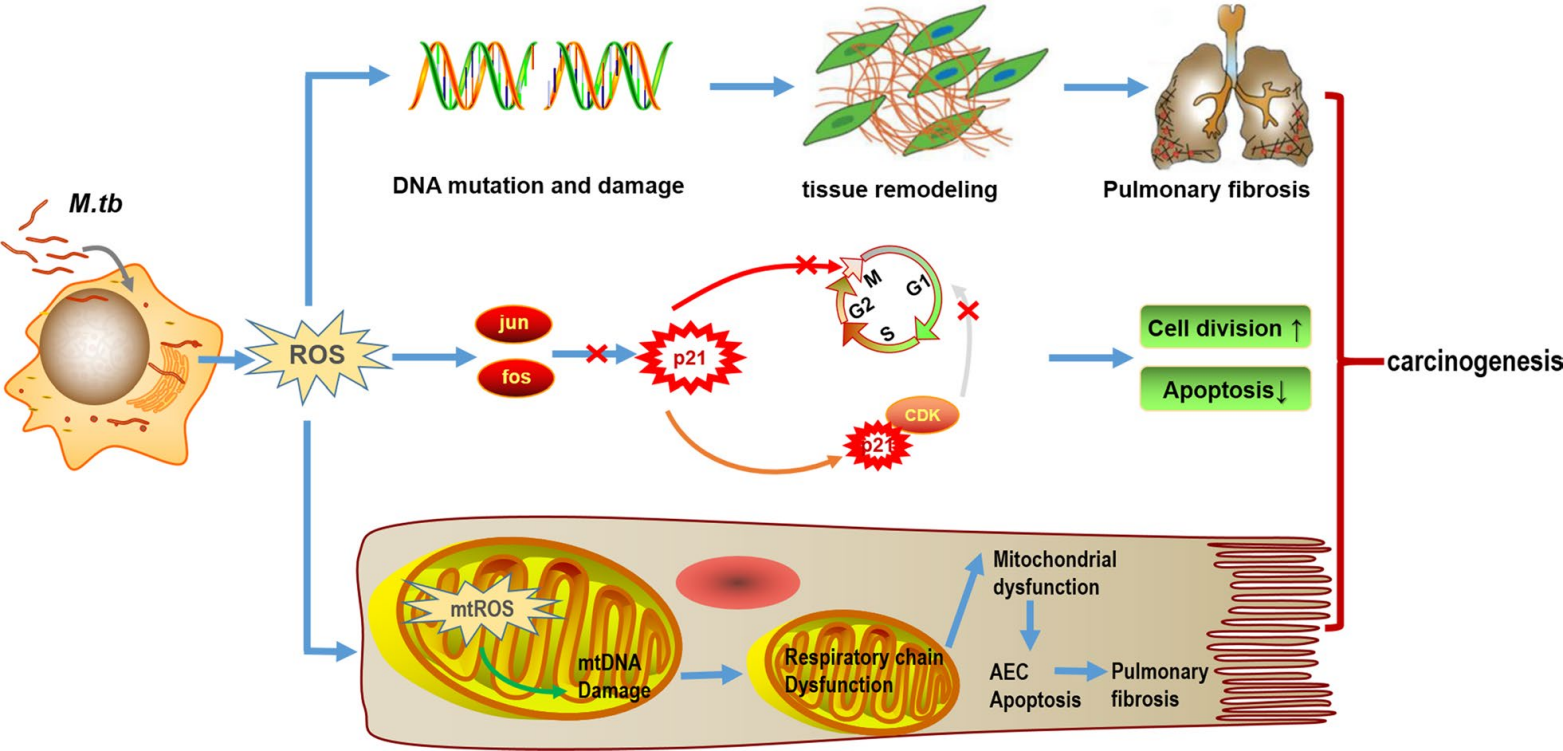
*M.tb* plays an important role in carcinogenesis in the lungs resulting from generation of reactive oxygen species (ROS) in the macrophage and alveolar epithelial cell. ROS can damage DNA and induce DNA mutation, that initiate tissue remodeling and pulmonary fibrosis, which promotes lung cancer. ROS also upregulate oncogenes jun and fos, which are involved in cell cycle progression through inhibition of P21, that promote cell division and inhibit apoptosis. In mitochondria, mtROS damages mtDNA and induce AEC apoptosis through interrupting respiratory chain and mitochondrial function, which leads to pulmonary fibrosis and lung cancer

*Mycobacterium tuberculosis* infection can upregulate reactive oxygen species (ROS) and nitric oxide in macrophages [56–59] and alveolar epithelial cells [60]. ROS is a key weapon used by macrophages to defend against pathogens, especially *Mycobacterium tuberculosis*, Phagocytes produce excessive ROS, which helps destroy *Mycobacterium tuberculosis* resident in phagosomes, as a key host defense mechanism [61]. The ROS produced by inflammatory cells in the microenvironment can cause chromosomal strand disruption and the accumulation of DNA mutations. Several studies have displayed that, ROS released by abnormal mitochondria can induce mitochondrial DNA (mtDNA) damage in the process of pulmonary fibrosis. The important role of ROS in aging, lung diseases including Idiopathic pulmonary fibrosis (IPF) and lung cancer is unquestionable [62]. ROS can oxidize a series of cellular targets, including mtDNA, lipids and proteins. These targets activate a series of biological processes, such as DNA damage response, mitochondrial dysfunction, inhibition of apoptosis and signal transduction limitation, leading to tissue damage, delaying wound healing and fibrosis [63–65]. In addition, the danger-associated molecular patterns (DAMPS) released by cells stimulated by ROS may be more related to IPF [66]. Studies report that mtDNA exposure is sufficient to activate macrophages and fibroblasts in the experimental environment to promote pulmonary fibrosis [67]. Under the circumstances of pulmonary fibrosis, mtDNA has involved in the formation of neutrophil extracellular traps that lead to inflammation and tissue remodeling [68]. Therefore, the acceleration of ROS secretion and mtDNA damage are the main characteristics of pulmonary fibrosis. In addition, increased release of ROS leads to increased expression of oncogenes jun and fos [69, 70]. The activation of jun/fos promotes cell proliferation by inhibiting the expression of P21, which further leads to G2/M cycle arrest and progression to mitosis [71, 72]. P21 also binds to cyclin-dependent kinases and is responsible for inhibiting the progression from G0 phase arrest to G1 phase and the progression from G1 phase to S phase [73]. These changes in the cell cycle will not only promote the cell division rate, but also shorten the DNA repair time, and at the same time reduce the time to initiate apoptosis, damage and existing mutations in those dividing cells with DNA damage, thereby greatly increasing the risk of carcinogenesis (Fig. 2).

## COX-2 of pulmonary tuberculosis and lung cancer

**Fig. 3:**
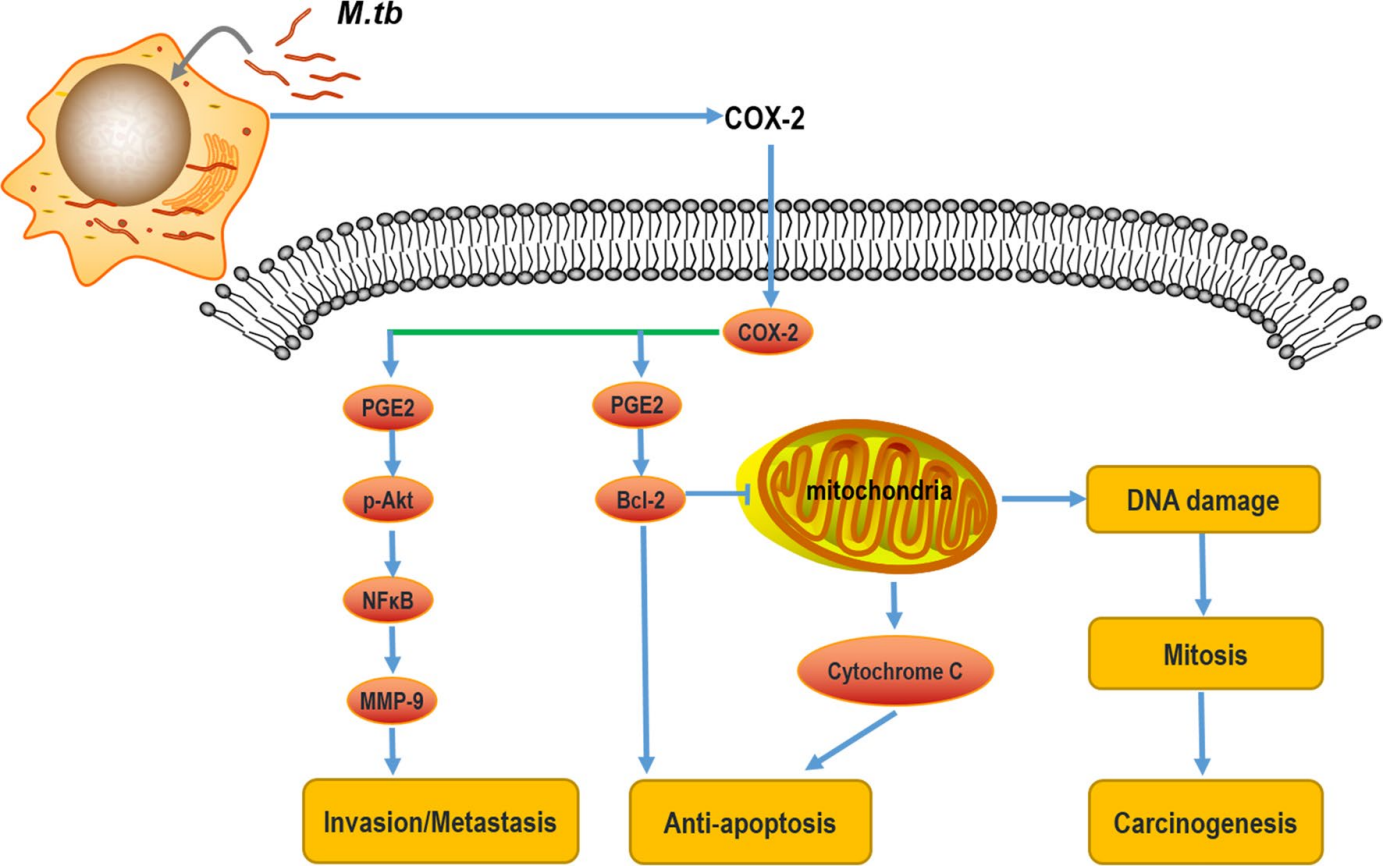
The role of COX-2 in tumorigenesis. COX-2 is induced in the DC cells and monocytes infected with *M.tb*. On the one hand, promotion of tumor metastasis of COX-2 depends on the p-Akt-NF-κB pathway affecting the activity of MMP-9. On the other hand, up-regulation of COX-2 can increase BCL-2 synthesis to inhibit apoptosis, and BCL-2 inhibits mitochondrial-mediated apoptosis pathway, mitochondrial dysfunction aggravates DNA damage, which further promotes cell mitosis and tumorigenesis

It has been determined previously that the overexpression of Prostaglandin E2/ Cyclooxygenase 2 (PGE2/COX-2) leads to pathogenesis in several bacterial, fungal, and viral infections, such as *Escherichia coli* [74, 75], *Streptococcus suis* [76], *Chlamydia trachomatis* [77], *Candida albicans* [78]. Correspondingly, recent studies showed that PGE2/COX-2 signal transduction was activated in the macrophages infected with *Mycobacterium tuberculosis*, which may lead to immune response dysfunction that provides the survival and replication niche for *Mycobacterium tuberculosis* [79]. Myeloid-derived suppressor cells (MDSC) are increased in the peripheral blood in those with active TB through upregulation of COX-2 and PGE2 expression. COX-2 inhibitors are also being evaluated for host-directed therapy (HDT) of tuberculosis patients [80]. Quantitative RT-PCR analysis and protein detection showed that PGE2/COX-2 signal transduction was activated, manifested by up-regulation of PGE2 expression and COX-2 and microsomal PGE2 synthase (mPGES) in DCs infected with *Mycobacterium* *Bovis* and BCG [81]. In monocyte subsets, there had basal expression of COX-2 and 5-LOX, and the expression increased significantly after stimulation with purified protein derivative (PPD) of *Mycobacterium tuberculosis* [82]. Recent evidence display that upregulation of COX-2 promotes tumorigenesis by promoting the metastasis signal affecting MMP-9 activity, it attenuates cancer cell migration and invasion via Akt, NF-Κb, and AP-1 signal pathways [83]. In addition to affecting tumor metastasis, upregulation of COX-2 is correlated with inhibition of apoptosis and increased intratumoral microvessels growth [84–86]. Inhibition of apoptosis is associated with enhanced BCL-2 synthesis, and this phenomenon is mediated by PGE-2 produced by COX-2. The increased synthesis of BCL-2 inhibits the cytochrome C-mediated apoptosis pathway, which in turn leads to more DNA damage. Cells with DNA mutations enter mitosis, thereby increasing the risk of cancer. All the relevant events discussed above indicate that tuberculosis patients have a higher risk of developing lung cancer by increasing DNA damage, inhibiting cell apoptosis, increasing cell division rate, and enhancing angiogenesis, these phenotypes can be initiated by COX-2 (Fig. 3).

## C-reactive protein of pulmonary tuberculosis and lung cancer

In fact, high serum C-reactive protein levels (CRP) (usually markers of inflammation) are risk factors for the development of lung cancer [87]. Another Chinese clinic study in Hangzhou found that elevated serum CRP levels will increase the incidence of lung cancer in male tuberculosis patients [88]. When the patient is in a state of chronic inflammation, inflammatory cells secrete IL-1, IL-6 and TNFα, which induce the liver to further secrete CRP. CRP activates lymphocytes, endothelial cells and smooth muscle cells, thereby promoting the expression of adhesion molecules and chemokines, thereby inducing cell carcinogenesis. Peroxidation in the process of continuous chronic inflammation can cause cell damage, causing gene mutations in the function of related coding proteins involved in gene repair, mutations in tumor suppressor genes, and inducing lung cell carcinogenesis [89].

## DNA damage of pulmonary tuberculosis and lung cancer

It is currently recognized that DNA damage directly or indirectly caused by mutagens and carcinogens is the main cause of chromosomal abnormalities. Interestingly, chronic TB infection in a mouse model has been shown to induce malignant and tumorigenic squamous cell aggregates in the lung, mediated by DNA damage and the production of epidermal growth factor (EGF) Epiregulin, this growth factor is a member of the epidermal growth factor family and as a ligand for the EGF receptor (EGFR), which can act as a paracrine survival and growth factor responsible for squamous metaplasia and tumorigenesis. The carcinogen formed after smoking tobacco burns promotes DNA damage and is closely related to the etiology of lung cancer [90–94]. This is a typical example. This has also become another important reason for the susceptibility of lung cancer in pulmonary tuberculosis patients with a history of smoking. Severe lung tissue damage mediated by the TB susceptibility locus sst1 is more significant in the occurrence of lung cancer caused by chronic TB infection [12]. Camilo Molina-Romero stated that *Mycobacterium tuberculosis* DNA can integrate into bronchial epithelial cells and induce lung neoplastic transformation [95]. In addition, Nalbandian et. al proved that *Mycobacterium tuberculosis* can hijack the host immune response, and modify the host local microenvironment to trigger a series of events that eventually conduct to tumor development. In a mouse model, persistent *Mycobacterium tuberculosis* infection is adequate to induce the multi-step transformation of cells related to tuberculosis to malignant squamous cell carcinoma through squamous cell dysplasia [33].

## Expression of inhibitory receptors in pulmonary tuberculosis and lung cancer

TB infection can aggravate chronic inflammation, which may not only weaken the innate and acquired immune responses, but may also be related to the abnormal expression of immune-associated genes [96]. Tuberculosis infection, chronic obstructive pulmonary disease (COPD), smoking and cooking fume exposure, are defined as chronic inflammation-associated environmental exposures, and the interaction between these exposure factors and single nucleotide polymorphisms (SNPs) in lung cancer susceptibility has also been highly valued by researchers [97]. In this study, tuberculosis infection was an environmental exposure related to the risk of lung adenocarcinoma in women who had never smoked. In addition, programmed death ligand 2 (PDCD1LG2) SNPs rs78096119 and rs12237624 are significantly related to tuberculosis infection, which is associated with lung cancer. This finding emphasizes the importance of gene-environment interactions for lung cancer in never-smokers. A new SNP for PDCD1LG2 related to lung adenocarcinoma risk has been identified. Among them, two SNPs related to susceptibility to lung adenocarcinoma are related to tuberculosis infection [97]. Cao et al. analyzed the expression levels and function of PD-1, PD-L1, and PD-L2 in antigen-specific T cells from *Mycobacterium tuberculosis* patients and spleen lymphocytes from wild type and PD-1 knockout mice, and Lewis mice injected with lung cancer cells. The results show that the expression levels of PD-1, PD-L1, and PD-L2 are elevated in patients with active tuberculosis and in mice treated with *Mycobacterium tuberculosis* and lung cancer cells. Moreover, *Mycobacterium tuberculosis* suppressed the cellular immune response mediated by T cells, while *Mycobacterium tuberculosis* significantly promoted lung cancer metastasis. In short, the PD-1/PD-L pathway is necessary for *Mycobacterium tuberculosis* to suppress T cell immune response and promote tumor metastasis. This research provided evidence that blocking the PD-1/PD-L1 signaling pathway may benefit patients with *Mycobacterium tuberculosis* or other chronic infections, and may even prevent them from developing cancer [98]. Multiple lines of evidence displayed that tuberculosis infection is accompanied by increased expression of immune inhibitory receptors in immune cells, such as NK cell and T cell. *M. tuberculosis/*Simian immunodeficiency virus (SIV)–coinfected animals had a higher frequency T cell immunoreceptor with immunoglobulin and ITIM domain (TIGIT) and PD-1 expression in CD4^+^ and CD8^+^ T cells [99]. TIGIT is an inhibitory receptor, which exerts a crucial role in limiting innate and adaptive immunity. The increased expression of TIGIT in T cells and NK cells were associated with impaired NK and T cells function, and promoted tumor immune escape [100]. NK cell dysfunction is present prior to tuberculosis infection. Loss of Mtb control allows the development of other related diseases [101]. The immunosuppressive function of tuberculosis by naturally occurring regulatory T cells may amplify the hazard of malignancy [102]. However, the causal relationship between the expression of inhibitory receptors and the occurrence of tuberculosis causing lung cancer remains to be further verified.

## Conclusions and perspectives

**Fig. 4:**
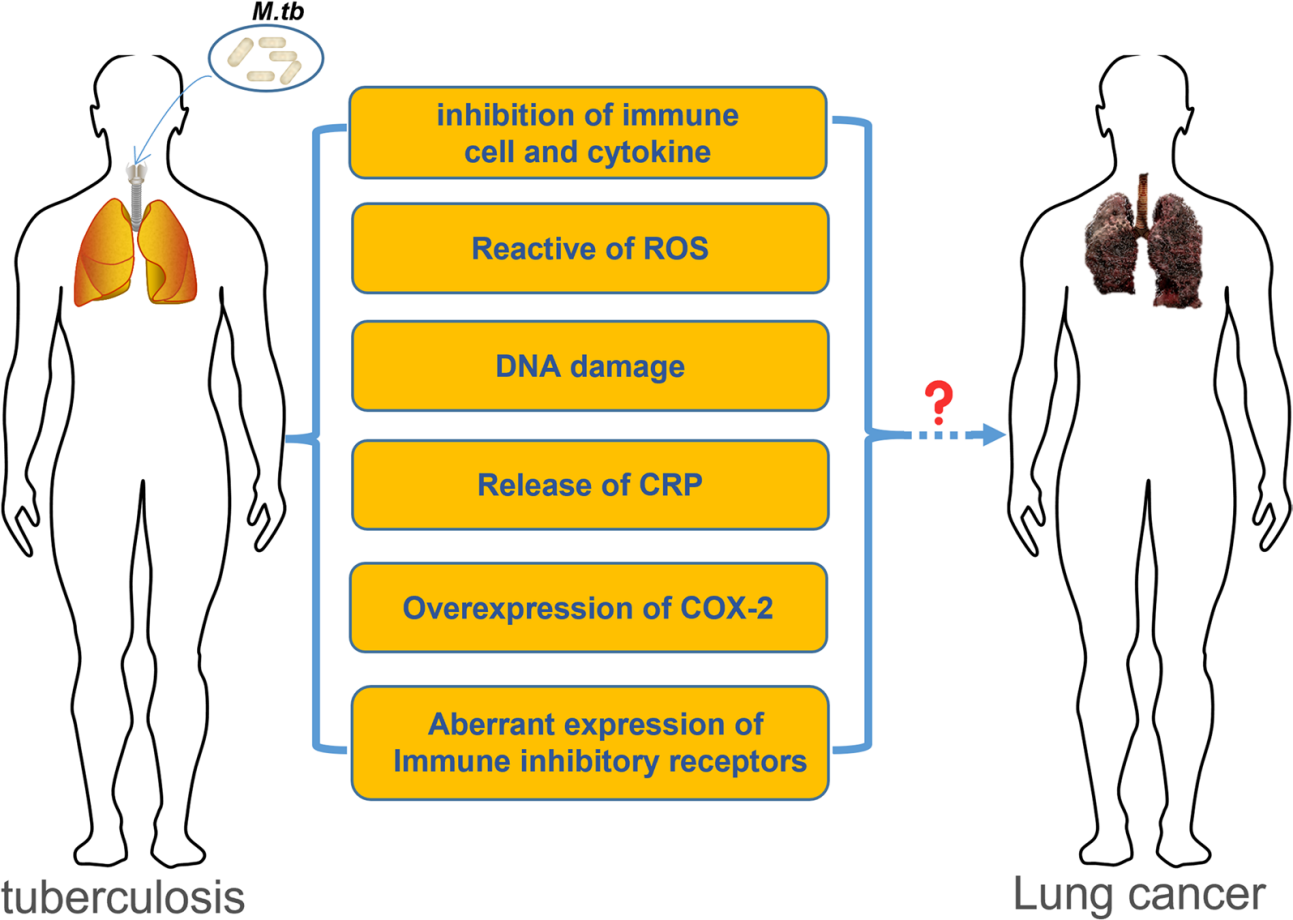
The relationship between tuberculosis and lung cancer

**Fig. 5:**
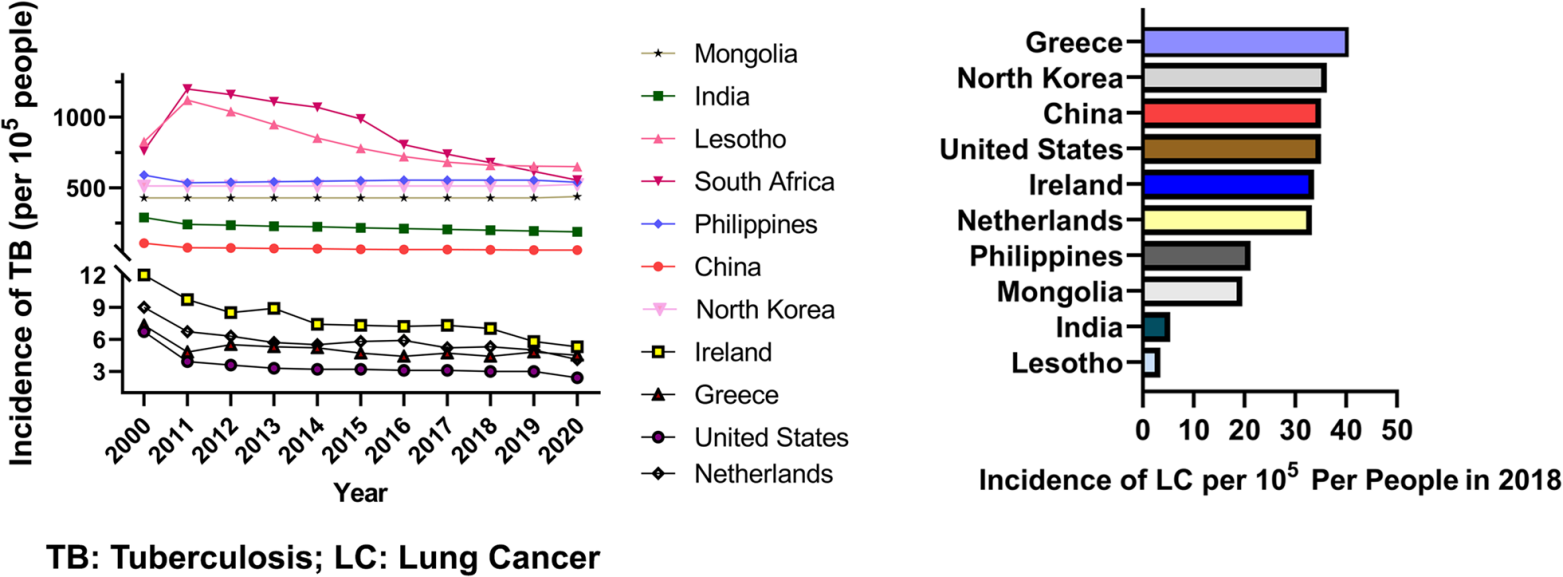
The incidence of tuberculosis and lung cancer in indicated countries

The epidemiology and molecular biology association between tuberculosis and lung cancer are well researched. Nevertheless, new molecular mechanisms are constantly being discovered. According to the WHO global burden of cancer in 2018 and the WHO global Tuberculosis Report, there are nearly 2.09 million new lung cancer cases and nearly 10.0 million new tuberculosis cases worldwide each year, both of which constitute the burden of tuberculosis-lung cancer. Therefore, in order to make an early and timely diagnosis, better prevention and effective treatment, and reduce the incidence and mortality of tuberculosis and lung cancer, it is of great significance to study the risk factors related to the development of tuberculosis into lung cancer (Fig. 4). In a cohort study of 1,936,512 patients, tuberculosis is correlated with a 1.67-fold increase in the risk of secondary lung cancer [10]. All complications may increase the risk of secondary lung cancer compared with the non-tuberculosis cohort in primary cancer. Some even believe that Mtb is an absolute lung carcinogen, Mtb acts as an initiator and promoter of tumor growth [103]. As a result, clinicians should consider this in patients with TB infection, because in patients with primary cancer, TB is more likely to cause secondary lung cancer [10]. Although a large number of research results indicate that tuberculosis and lung cancer may be anatomically close and develop in the same anatomical unit in the lung, however, some scholars hold the opposite view that this does not indicate that there is clear pathogenesis between tuberculosis and lung cancer. The relationship between tuberculosis and lung cancer is far more complicated than existing research results, and does not conform to a simple causal relationship scheme [104]. We compared the incidence of lung cancer in countries with a high and low incidence of tuberculosis (data source: https://databank.worldbank.org/reports.aspx?source=2&series=SH.TBS.INCD&country=#; https://stat.link/gc07yo). It is displayed that in countries with low tuberculosis incidence, their incidence of lung cancer has not decreased (Fig. 5). And countries like Lesotho, with a high incidence of tuberculosis, do not have a high incidence of lung cancer, while countries like Korea have a high incidence of tuberculosis and a high incidence of lung cancer. It is worthy to note that, our statistics about the incidence of lung cancer are limited to 2018. Therefore, tuberculosis can increase the risk of lung cancer, but tuberculosis is not more than a direct risk factor for lung cancer, the occurrence of lung cancer is the result of multiple factors.

On the whole, although there is not much definite evidence that pulmonary tuberculosis will definitely develop into lung cancer, multiple lines of studies have shown that chronic inflammation, immune regulation imbalance, and gene mutation caused by tuberculosis are related to the occurrence of lung cancer. On the one hand, this review hopes that clinicians can give relevant assessments on whether tuberculosis patients are likely to develop lung cancer, personalized medicine for the management, and provide early intervention. On the other hand, it also hopes that researchers will pay more attention to the underlying pathogenesis between tuberculosis and lung cancer in the future basic research. Clarifying the causal relationship between tuberculosis and lung cancer has a long way to go.

## Data Availability

All data generated or analyzed during this study are included in this published article.

## Abbreviations

COX-2: Cyclooxygenase 2
DAMPS: Danger-Associated Molecular Patterns
HDT: Host-Directed Therapy
ICI: Immune Checkpoint Inhibitors
IPF: Idiopathic pulmonary fibrosis
KO: Knock-Out
*M.tb*: *Mycobacterium tuberculosis*
MDSC: Myeloid-Derived Suppressor Cells
mtDNA: Mitochondrial DNA
NOX4: NADPH Oxidase 4
PPD: Purified Protein Derivative
PGE2: Prostaglandin E2
SNP: Single Nucleotide Polymorphisms
TB: Tuberculosis
VEGF: Vascular Endothelial Growth Factor
